# Structural Insights into the Mechanisms of Action of Functionally Distinct Classes of Chikungunya Virus Nonstructural Protein 1 Inhibitors

**DOI:** 10.1128/AAC.02566-20

**Published:** 2021-06-17

**Authors:** Kristina Kovacikova, Marina Gorostiola González, Rhian Jones, Juan Reguera, Alba Gigante, María-Jesús Pérez-Pérez, Gerhard Pürstinger, Julia Moesslacher, Thierry Langer, Lak Shin Jeong, Leen Delang, Johan Neyts, Eric J. Snijder, Gerard J. P. van Westen, Martijn J. van Hemert

**Affiliations:** a Department of Medical Microbiology, Leiden University Medical Center, Leiden, the Netherlands; b Division of Drug Discovery and Safety, Leiden Academic Centre for Drug Research, Leiden University, Leiden, the Netherlands; c Oncode Institute, Leiden, the Netherlands; d Aix-Marseille Université, INSERM, CNRS, AFMB UMR 7257, Marseille, France; e Instituto de Química Médica (IQM-CSIC), Madrid, Spain; f Department of Pharmaceutical Chemistry, University of Innsbruck, Innsbruck, Austria; g Department of Pharmacy, University of Innsbruck, Innsbruck, Austria; h Department of Pharmaceutical Chemistry, University of Vienna, Vienna, Austria; i College of Pharmacy, Seoul National University, Seoul, South Korea; j KU Leuven, Department of Microbiology, Immunology and Transplantation, Rega Institute for Medical Research, Laboratory of Virology and Chemotherapy, Leuven, Belgium

**Keywords:** Chikungunya virus, antivirals, nsP1, capping, resistance, molecular docking, SAM, GTP, binding pocket, inhibitors

## Abstract

Chikungunya virus (CHIKV) nonstructural protein 1 (nsP1) harbors the methyltransferase (MTase) and guanylyltransferase (GTase) activities needed for viral RNA capping and represents a promising antiviral drug target. We compared the antiviral efficacies of nsP1 inhibitors belonging to the MADTP, CHVB, and FHNA series (6′-fluoro-homoneplanocin A [FHNA], its 3′-keto form, and 6′-β-fluoro-homoaristeromycin). Cell-based phenotypic cross-resistance assays revealed that the CHVB and MADTP series had similar modes of action that differed from that of the FHNA series. In biochemical assays with purified Semliki Forest virus and CHIKV nsP1, CHVB compounds strongly inhibited MTase and GTase activities, while MADTP-372 had a moderate inhibitory effect. FHNA did not directly inhibit the enzymatic activity of CHIKV nsP1. The first-of-their-kind molecular-docking studies with the cryo-electron microscopy (cryo-EM) structure of CHIKV nsP1, which is assembled into a dodecameric ring, revealed that the MADTP and CHVB series bind at the *S*-adenosylmethionine (SAM)-binding site in the capping domain, where they would function as competitive or noncompetitive inhibitors. The FHNA series was predicted to bind at the secondary binding pocket in the *r*ing-*a*perture *m*embrane-*b*inding and *o*ligomerization (RAMBO) domain, potentially interfering with the membrane binding and oligomerization of nsP1. Our cell-based and enzymatic assays, in combination with molecular docking and mapping of compound resistance mutations to the nsP1 structure, allowed us to group nsP1 inhibitors into functionally distinct classes. This study identified druggable pockets in the nsP1 dodecameric structure and provides a basis for the rational design, optimization, and combination of inhibitors of this unique and promising antiviral drug target.

## INTRODUCTION

Chikungunya virus (CHIKV) and Semliki Forest virus (SFV) are Old World alphaviruses belonging to the *Togaviridae* family. This group includes mosquito-borne enveloped viruses with single-stranded, positive-sense RNA genomes of approximately 12 kb. CHIKV is an arthritogenic alphavirus transmitted by the Aedes aegypti and Aedes albopictus mosquitoes. Infections with Old World alphaviruses such as CHIKV typically result in symptoms such as fever, rash, and polyarthritis/polyarthralgia. In roughly two-thirds of cases, CHIKV infection progresses into a severe form of persistent, debilitating joint pain with long-term sequelae (1). Since its reemergence in Kenya in 2004 and its introduction into new territories in Asia, the Caribbean, the Americas, and southern Europe, CHIKV has infected millions of people worldwide (2). Despite recent advances in CHIKV vaccine development (3), prophylactic and/or therapeutic treatment for CHIKV infection is still lacking.

Alphavirus nonstructural proteins (nsPs) are released from a polyprotein precursor by proteolytic cleavage and are indispensable in the alphaviral life cycle (4). The viral replication complex, formed by nsP1 to -4, assembles in intracellular compartments, termed spherules, which are derived from the host plasma membrane (5, 6). nsP1 is responsible for the membrane association of the replication complex (7, 8). In addition, nsP1 catalyzes viral RNA capping, whereby the 5′ end of the nascent RNA is modified by the attachment of a cap-0 (m^7^GpppA) structure. Capping of alphavirus RNA is an essential step in the replication cycle, since the cap structure protects viral mRNA from cellular exonucleases, enables its efficient translation, and prevents recognition by the host innate immune system. Alphaviruses use a mechanism of mRNA capping that is distinct from that of the host cell. While cellular capping enzymes methylate GTP after it has been transferred to the 5′ end of the RNA, alphavirus nsP1 first methylates GTP, after which the methylated GTP (m^7^GTP) is covalently attached to nsP1 and subsequently transferred to RNA (9). In the first step of the alphaviral capping reaction sequence, *S*-adenosylmethionine (SAM)-dependent N7-methyltransferase (MTase) methylates a GTP molecule while releasing *S*-adenosylhomocysteine (SAH) as a by-product (10, 11). In the second step, the guanylyltransferase (GTase) activity of nsP1 mediates the attachment of m^7^GTP to nsP1 to form the covalent m^7^GMP-nsP1 intermediate, with release of pyrophosphate (PP_i_) (12). In the final reaction step, m^7^GMP is transferred onto the modified 5′ end of the viral mRNA. Before this event, the RNA 5′ triphosphatase activity of nsP2 removes the 5′-terminal γ-phosphate from triphosphorylated viral RNAs to yield 5′ diphosphate RNAs that can serve as substrates for the transfer of m^7^GMP from m^7^GMP-nsP1, resulting in the formation of a cap-0 structure (13). Early sequence analyses predicted that the N-terminal domain of alphavirus nsP1 (approximately 200 amino acids [aa]) contains a Rossmann fold with conserved sequence motifs, referred to as the “Core” region, and harbors the MTase activity (14). Later predictions suggested that the putative alphavirus MTase-GTase domain corresponds to the Core region and a C-terminal extension from aa 250 to approximately aa 406, termed the “Iceberg” region (15). Enzymatic assays with truncated SFV nsP1 indicated that the first 500 aa are required for full enzymatic activity (16). Despite knowledge obtained from mutagenesis studies with recombinant SFV nsP1, the binding sites for the endogenous ligands SAM and GTP are not currently known. A Sindbis virus (SINV) nsP1 mutant resistant to low intracellular GTP levels harbors three mutations in nsP1, namely, glutamine-21-lysine (Q21K), serine-23-asparagine (S23N), and valine-302-methionine (V302M) (17), indicating that a potential GTP-binding site might include residues from both the N-terminal and Iceberg regions of the protein. Furthermore, it is known that specific mutations and deletions within the MTase-GTase domain of alphavirus nsP1 abolish enzymatic activity and yield nonviable viruses (16, 18). For example, the SFV nsP1 aspartic acid-64-alanine (D64A) mutant was unable to bind SAM in a UV cross-linking assay, and this mutation interfered with MTase activity. Furthermore, the SFV nsP1 histidine-38-alanine (H38A) mutation selectively destroyed GTase activity, presumably by abolishing the covalent attachment of m^7^GMP to nsP1 (16). Besides mutational analysis, little progress has been made with regard to the understanding and characterization of alphavirus nsP1 functional domains due to the lack of a crystal structure that could provide insight into the spatial organization of various functional residues.

**FIG 1:**
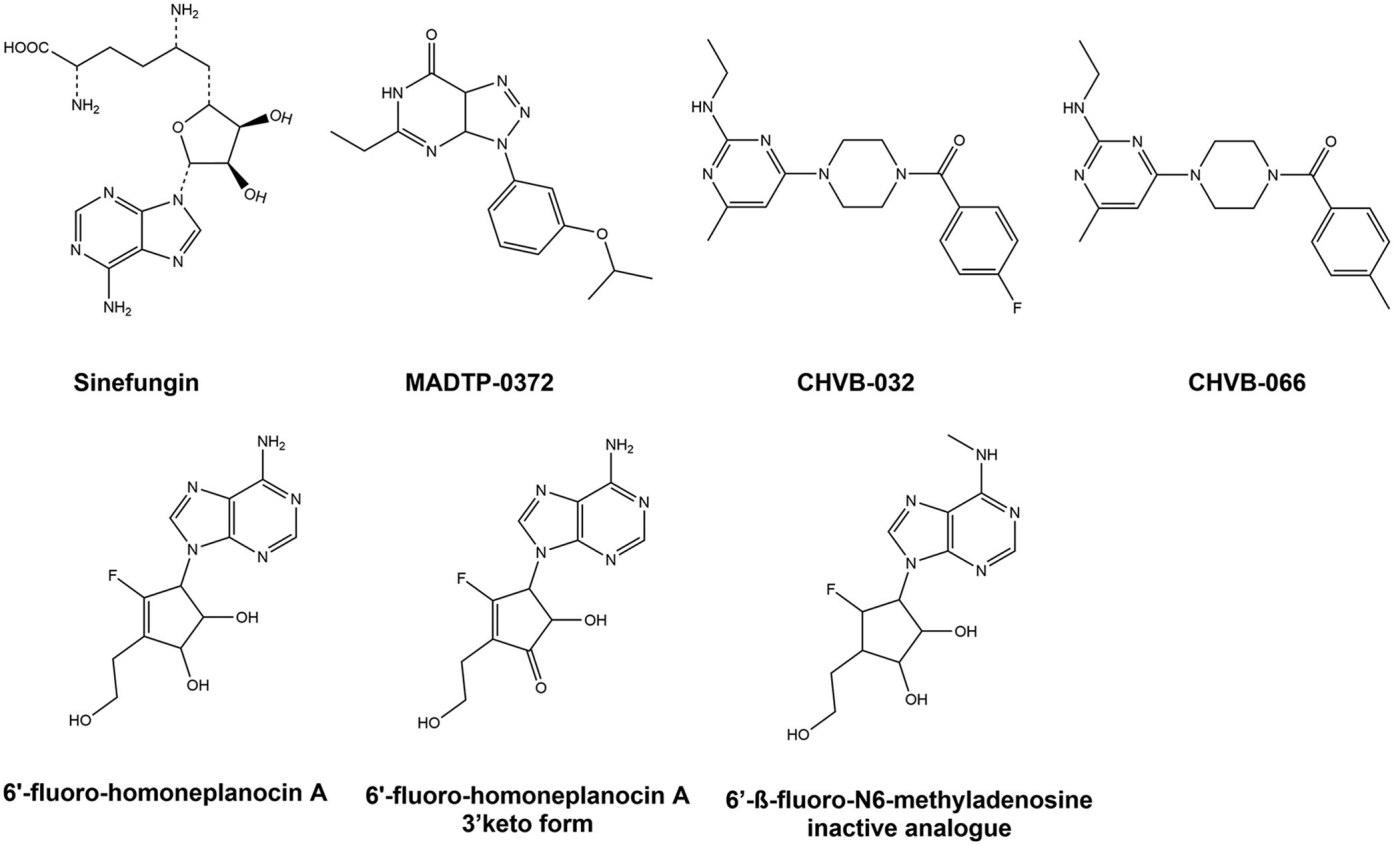
Chemical structures of the CHIKV nsP1-targeting compounds used in this study.

Several CHIKV nsP1 inhibitors have been discovered in recent years (reviewed in reference 19), indicating that alphavirus nsP1 is a promising antiviral drug target. MADTP-372 is a compound belonging to the 3-aryl-[1,2,3]triazolo[4,5-*d*]pyrimidin-7(6*H*)-ones, or MADTP series (20). Resistance selection and genotyping showed that a proline-34-serine (P34S) substitution in the N-terminal part of CHIKV nsP1 results in resistance to MADTP compounds (21). A threonine-246-alanine (T246A) substitution was also identified as a MADTP resistance mutation (unpublished data). 6′-β-Fluoro-homoaristeromycin (FHA) and 6′-fluoro-homoneplanocin A (FHNA) are carbocyclic adenosine analogues with potent anti-CHIKV activity, originally designed as SAH hydrolase inhibitors (22). CHIKV mutants carrying the glycine-230-arginine (G230R) and lysine-299-glutamic acid (K299E) substitutions in nsP1 are resistant to these compounds (22). CHVB-032 and CHVB-066 belong to 2-(4(phenylsulfonyl)piperazine-1-yl)pyrimidine analogues, also known as the CHVB series (23). Selection of resistant variants and reverse genetics studies indicated that the combined serine-454-glycine (S454G) and tryptophan-456-arginine (W456R) substitutions in the C-terminal part of CHIKV nsP1 are (primarily) responsible for CHVB resistance (24). Sinefungin is a SAM analogue that inhibits Venezuelan equine encephalitis virus (VEEV) and CHIKV nsP1 in enzymatic assays measuring either MTase or GTase activity (25–27). Besides sinefungin, MADTP-372 and CHVB-066 have been shown to inhibit the GTase activity of VEEV nsP1 in enzymatic assays (21, 24). We have shown previously that CHVB compounds completely block *in vitro* activity of SFV nsP1 in a covalent m^7^GMP-nsP1 complex formation assay (24), and we now report a similar, though less potent, effect for the MADTP series. Here, we set out to explore the cross-functional relationships between the various CHIKV nsP1 inhibitors (Fig. 1). We aimed to investigate whether these compounds have similar mechanisms of action, by, for example, sharing a binding pocket, and whether this could be linked to a function, e.g., interfering with a SAM- or GTP-binding site. To this end, we compared the inhibitors in cell-based cross-resistance assays and enzymatic assays, and we performed molecular docking on the recently solved CHIKV nsP1 cryo-electron microscopy (cryo-EM) structure (28).

## RESULTS

### Cross-resistance analysis of CHIKV nsP1 mutants resistant to different nsP1-targeting compounds.

**FIG 2:**
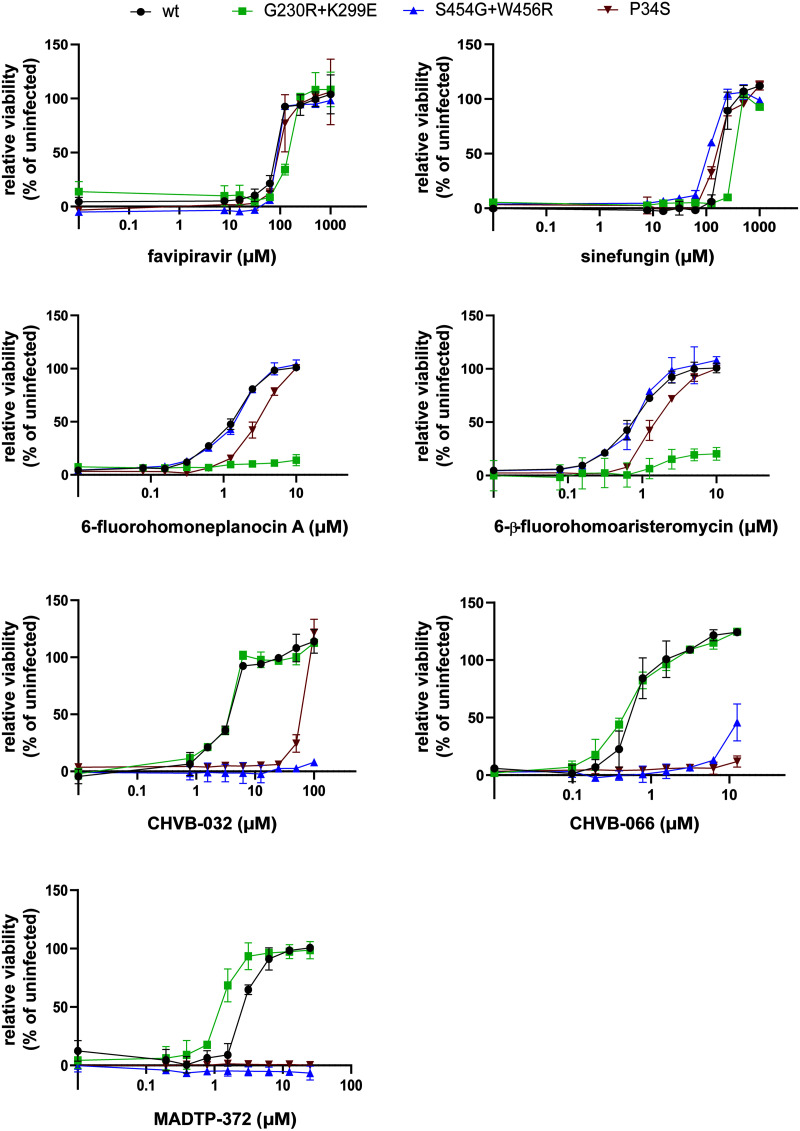
Cross-resistance analysis of compound-resistant CHIKV strains with mutations in nsP1. The sensitivities of wt CHIKV and the nsP1 G230R K299E, S454G W456R, and P34S mutants to various nsP1-targeting inhibitors were assessed in CPE reduction assays in Vero E6 cells. The mechanistically unrelated compound favipiravir, which targets nsP4, was included as a control. Data are expressed as cell viability relative to the viability of uninfected cells and represent means ± standard deviations from at least two independent experiments performed in quadruplicate (*n* = 8).

The anti-CHIKV activities of a variety of nsP1-targeting compounds, i.e., CHVB-032, CHVB-066, MADTP-372, FHA, FHNA, and sinefungin, were compared in a multicycle cytopathic effect (CPE) reduction assay on Vero E6 cells (Table 1). FHA and CHVB-066 were the most potent compounds, with 50% effective concentrations (EC_50_) below 1 μM. FHNA, CHVB-032, and MADTP-372 inhibited CHIKV with EC_50_ in the low micromolar range (1.2 to 3.4 μM). Sinefungin was not a potent inhibitor of CHIKV replication in cell culture; its EC_50_ was 184.9 μM. None of the compounds was cytotoxic at the effective concentrations. Furthermore, the same compounds were tested against SFV in the same type of multicycle infection assay. Interestingly, only FHA and FHNA inhibited SFV replication with EC_50_ in the low micromolar range (3.9 to 5.2 μM); the rest of the compounds did not exhibit any antiviral effect against SFV. Next, the CHIKV nsP1 mutant carrying the P34S substitution (resistant to MADTP-372) (21), the CHIKV nsP1 mutant carrying the G230R and K299E substitutions (resistant to FHA and FHNA) (22), and the CHIKV nsP1 mutant carrying the S454G and W456R substitutions (resistant to CHVB-032 and CHVB-066) (24) were tested in cross-resistance phenotypic assays in all possible compound-virus combinations and against the unrelated compound favipiravir (Fig. 2). All resistant mutants were sensitive to favipiravir, a nucleoside analogue that inhibits the CHIKV nsP4 RNA-dependent RNA polymerase (29), and sinefungin, a SAM analogue. CHIKV nsP1-P34S, which was originally selected as a MADTP-resistant virus, was completely resistant to MADTP-372 and to CHVB-066 at the concentrations tested, and it was >14-fold more resistant to CHVB-032 than the wild-type (wt) virus (Fig. 2). Furthermore, CHIKV nsP1-S454G+W456R, originally identified as a CHVB-resistant virus, was completely resistant to CHVB-032 and was cross-resistant to MADTP-372 at the doses tested and, to a lesser extent, to CHVB-066 (Fig. 2). This suggested that the compounds of the CHVB and MADTP series have similar mechanisms of action. CHIKV nsP1-G230R+K299E was resistant to FHA and FHNA but was sensitive to the CHVB and MADTP series (Fig. 2), suggesting a different mechanism of action that is unrelated to that of the CHVB and MADTP series. The comparison of fold resistance values, determined as the ratio of the EC_50_ for a resistant CHIKV mutant to that for wt CHIKV, for each resistant-mutant–compound combination is included in Table 2.

**TABLE 1:** Antiviral activities of nsP1 inhibitors against CHIKV and SFV replication in CPE reduction assays

Compound	CHIKV		SFV	
	EC_50_ (μM)^*a*^	CC_50_ (μM)^*b*^	EC_50_ (μM)	CC_50_ (μM)
FHA	0.7 ± 0.08	>250	3.9 ± 3.5	>250
FHNA	1.2 ± 0.03	>250	5.2 ± 3.2	>250
CHVB-066	0.6 ± 0.1	25	NA^*c*^	25
CHVB-032	3.4 ± 0.3	>100	NA	>100
MADTP-372	2.7 ± 0.2	>100	NA	>100
Sinefungin	184.9 ± 38.4	>1,000	NA	>1,000

a EC_50_, the concentration of a compound that reduces virus-induced CPE by 50%.

b CC_50_, the concentration of a compound that reduces cell viability by 50%.

c NA, not active.

**TABLE 2:** Resistance and cross-resistance of CHIKV nsP1 compound-resistant mutants against CHIKV nsP1 inhibitors and against a mechanistically unrelated compound, favipiravir

Compound	EC_50_ for wt CHIKV
		rCHIKV P34S		rCHIKV G230R + K299E		rCHIKV S454G + W456R	
		EC_50 (μM)_	Fold resistance^*a*^	EC_50 (μM)_	Fold resistance	EC_50 (μM)_	Fold resistance
FHA	0.7 ± 0.08	1.6 ± 0.2	2.3	>10	14.3	0.7 ± 0.08	1
FHNA	1.2 ± 0.03	2.8 ± 0.3	2.4	>10 (8.6)	8.6	1.2 ± 0.1	1
CHVB-066	0.6 ± 0.1	>12.5	20.8	0.3 ± 0.04	<1	>12.5	20.8
CHVB-032	3.4 ± 0.3	52.4 ± 0.8	14.4	3.4 ± 0.2	1	>100	29.4
MADTP-372	2.7 ± 0.2	>25	9.3	1.2 ± 0.2	<1	>25	9.3
Sinefungin	184.9 ± 38.4	150.8 ± 2.5	<1	274.5 ± 0.2	1.5	109.8 ± 3.9	<1
Favipiravir	79.8 ± 1.4	95.9 ± 12.8	1.2	135 ± 5	1.7	89.5 ± 0.2	1.1

a Calculated as (EC_50_ for the variant)/(EC_50_ for the wt).

### Inhibition of alphavirus enzymatic activity by nsP1-targeting compounds.

**FIG 3:**
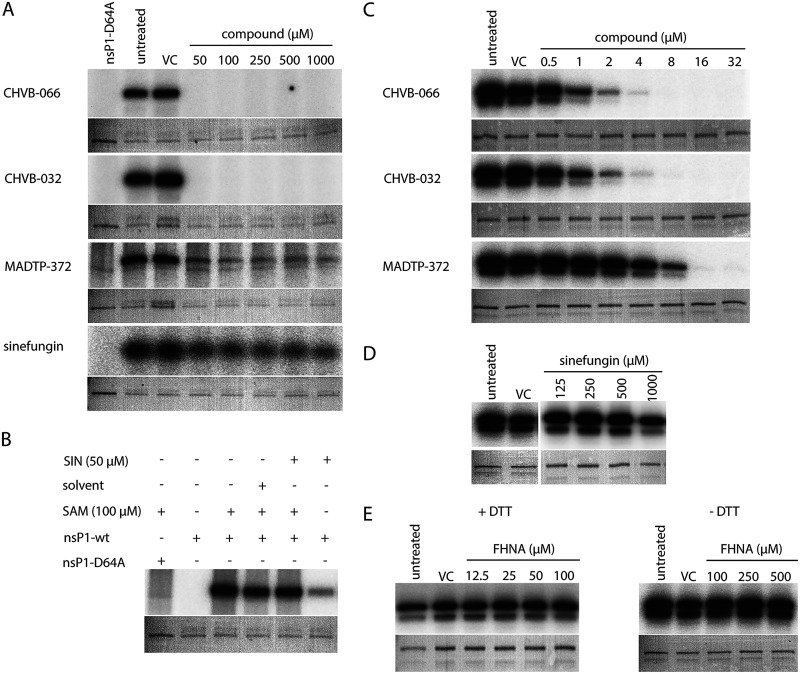
Inhibitory effects of selected compounds on the enzymatic activity of purified wt SFV or CHIKV nsP1 in a biochemical assay measuring the formation of the covalent [^32^P]m^7^GMP-nsP1 reaction intermediate. (A) wt SFV nsP1 was incubated with [α-^32^P]GTP and 100 μM SAM and was treated with increasing doses (50 μM to 1 mM) of inhibitors. SFV nsP1 D64A was used as a negative control. VC, solvent control. (B) wt SFV nsP1 was incubated with [α-^32^P]GTP with or without 50 μM sinefungin (SIN) in the presence or absence of 100 μM SAM. SFV nsP1 D64A was used as a negative control. (C) wt CHIKV nsP1 was incubated with [α-^32^P]GTP and 10 μM SAM and was treated with increasing doses (0.5 to 32 μM) of inhibitors. (D) wt CHIKV nsP1 was incubated with [α-^32^P]GTP and 10 μM SAM and was treated with increasing doses (125 μM to 1 mM) of sinefungin. (E) wt CHIKV nsP1 was incubated with [α-^32^P]GTP and 10 μM SAM in the presence (left) or absence (right) of DTT and increasing concentrations (12.5 to 500 μM) of FHNA. In all cases, the covalent [α-^32^P]m^7^GMP-nsP1 intermediate was visualized after overnight exposure of the PhosphorImager screen. Coomassie blue staining with GelCode blue reagent was used to demonstrate the loading of equal protein quantities.

We next determined the inhibitory effects of sinefungin, MADTP-372, CHVB-032, and CHVB-066 in an enzymatic assay monitoring the formation of the covalent m^7^GMP-nsP1 complex with either purified wt SFV or CHIKV nsP1. It should be emphasized that wt SFV nsp1 was purified from Escherichia coli (22) in an unknown conformation, while active oligomeric wt CHIKV nsP1 was produced using a baculovirus expression system (28). The covalent-complex formation assay measures both MTase and GTase activities and uses the formation of radioactive [^32^P]m^7^GMP-nsP1 as a readout (22). The active-site mutant SFV nsP1 D64A was used as a negative control, because it is devoid of MTase activity (16). CHVB-032 and its analogue CHVB-066 completely blocked the formation of the covalent [^32^P]m^7^GMP-nsP1 complex in an assay containing SFV nsP1 (Fig. 3A), as published previously (24). MADTP-372 also inhibited the formation of the covalent [^32^P]m^7^GMP-nsP1 intermediate, but less effectively; some radioactive signal was still detected even at a 1 mM dose of the compound (Fig. 3A). Sinefungin, on the other hand, was a very inefficient inhibitor, inducing a slight and steady reduction of signal across the concentration range tested (50 μM to 1 mM) (Fig. 3A). Sinefungin appeared to stimulate GTase activity in an assay with SFV nsP1, since some product was formed in reactions lacking SAM but containing 50 μM sinefungin (Fig. 3B). FHNA (not shown) inhibited the formation of the covalent [^32^P]m^7^GMP-nsP1 intermediate to a lesser extent in a modified SFV nsP1 covalent-complex formation assay lacking dithiothreitol (DTT) (22). In the assays containing CHIKV nsP1, CHVB-032, its analogue CHVB-066, and MADTP-372 inhibited the formation of the covalent [^32^P]m^7^GMP-nsP1 intermediate in a dose-dependent manner with increasing drug concentrations (0.5 to 32 μM) (Fig. 3C). In contrast, treatment with high concentrations (125 to 500 μM) of sinefungin did not lead to any reduction in the signal; only treatment with a 1 mM dose reduced the signal slightly from that of the untreated control (Fig. 3D). This might explain why sinefungin was inactive in a CPE reduction assay with SFV and exhibited a high EC_50_ in a similar assay with CHIKV (Table 1). Lastly, we aimed to evaluate whether FHNA would exert an inhibitory effect in the CHIKV nsP1 covalent complex formation assay in the presence or absence of DTT. Surprisingly, there was no reduction in the signal in assays with or without DTT (Fig. 3E), suggesting that FHNA does not directly inhibit the enzymatic activity of the oligomeric CHIKV nsP1. Unfortunately, the 3′-keto form of FHNA, which is suspected to be the active form of the molecule (22) could not be tested, because we were unable to synthesize this molecule. The combined data from the cell-based cross-resistance analysis and enzymatic assays with purified wt SFV or CHIKV nsP1 suggested that the various compounds can be divided into distinct classes based on their modes of action. Therefore, we set out to perform molecular docking studies with the individual nsP1 inhibitors and the CHIKV nsP1 cryo-EM structure, in order to understand the structural basis of nsP1 inhibition and to obtain more insights into the potential mechanisms of action of these compounds at the molecular level.

### Predicted CHIKV nsP1 binding pockets and poses of endogenous ligands.

**FIG 4:**
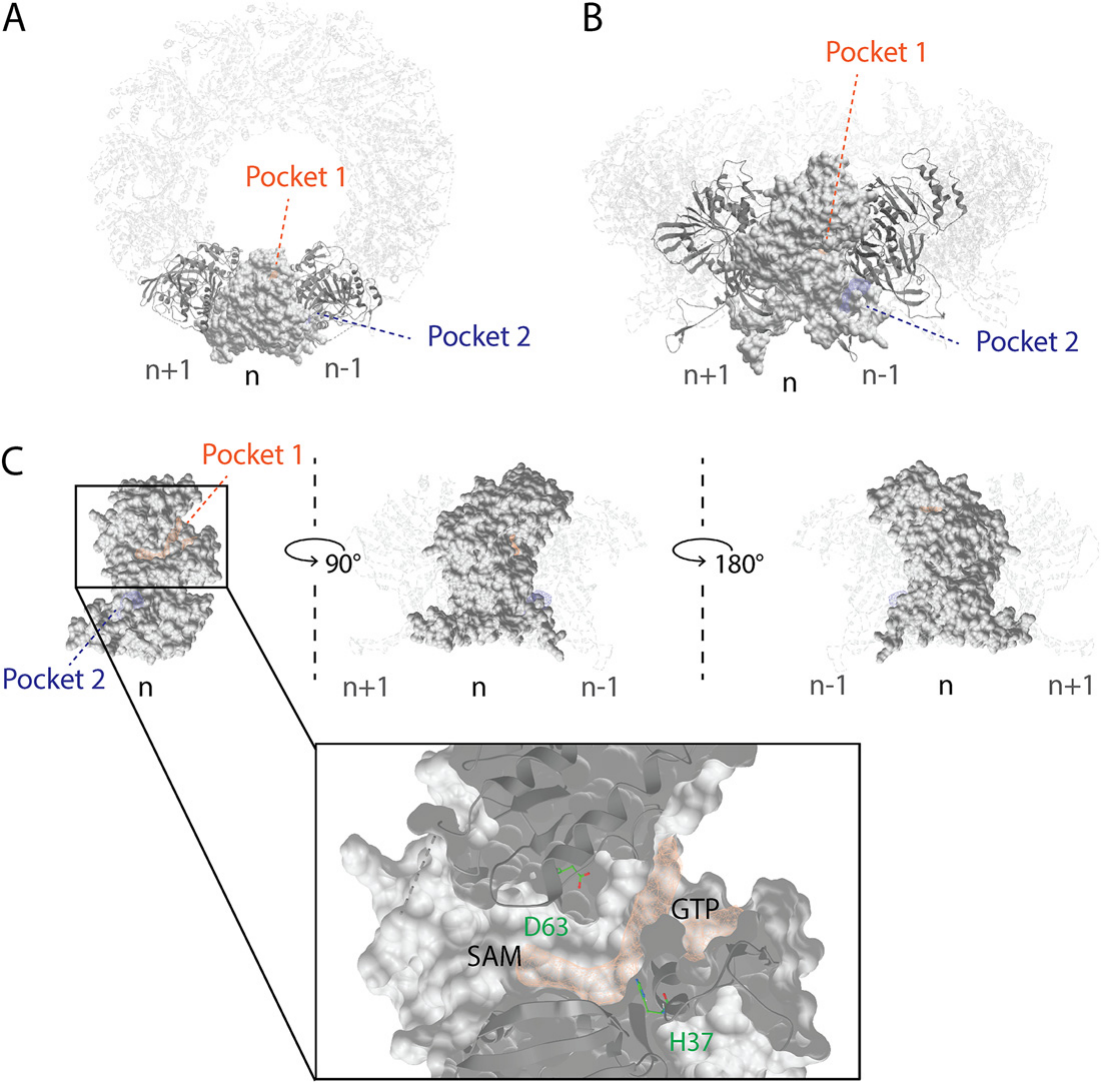
Predicted binding pockets in the CHIKV nsP1 oligomeric structure. (A) Top view of the nsP1 dodecameric ring. Shown are the main binding pocket (pocket 1, in orange) and secondary binding pocket (pocket 2, in blue) predicted by the ICM Pocket Finder method. (B) Front view of the nsP1 dodecameric ring. (C) The predicted main binding pocket is elongated and contains two binding sites virtually divided by the catalytic residues H37 and D63, which correspond to the SAM- and GTP-binding sites. Three consecutive nsP1 protomers, with subscripts n, n+1, and n–1, were used to define these pockets within the nsP1 complex (PDB code 6Z0V).

**FIG 5:**
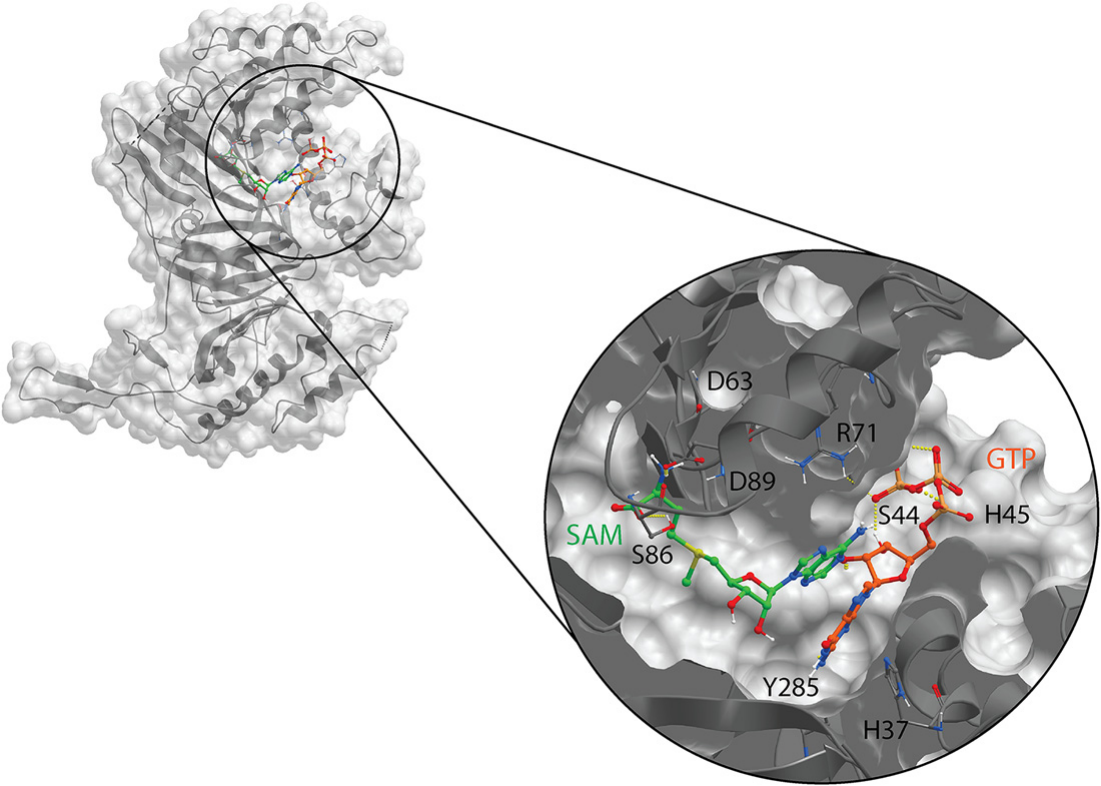
Suggested binding mode of the endogenous ligands GTP (orange) and SAM (green) in the catalytic pocket of CHIKV nsP1 (gray). The known catalytic residues H37 and D63 are not directly involved in binding, most likely due to the available conformation of the CHIKV nsP1 cryo-EM structure (PDB code 6Z0V), where D63 is deeply buried in the protein structure.

Using the ICM Pocket Finder method (30, 31) and the available CHIKV nsP1 cryo-EM structure representing a dodecameric ring (Fig. 4A and B), we identified an elongated ligand binding site, referred to as pocket 1 or the main binding pocket, which is depicted in Fig. 4. This pocket is part of the capping domain of nsP1, carrying out the MTase and GTase functions. We further recognized two binding sites in this predicted main binding pocket, which are virtually separated by H37 and D63, and which correspond to the GTP- and SAM-binding sites, as defined previously (28). A secondary binding pocket, referred to as pocket 2 in Fig. 4, was identified in the *r*ing-*a*perture *m*embrane-*b*inding and *o*ligomerization (RAMBO) domain of nsP1, which is involved in the membrane binding and oligomerization of nsP1 protomers (28). Based on secondary-structure predictions and cross-linking studies with recombinant SFV nsP1 (16), the catalytic site of CHIKV nsP1 is most likely flanked by H37 and D63. The endogenous ligands, GTP and SAM, are expected to bind in the proximity of these residues, as depicted in Fig. 5. However, in the available apo CHIKV nsP1 cryo-EM structure, the D63 residue, which is thought to be the anchor point for SAM (16), is too deeply buried in the protein structure to be accessible to endogenous ligands in docking simulations. Residue H37 is part of the catalytic loop in the active site, defined as a 4-aa region between residues P34 and H37. We hypothesize that a conformational change takes place during the capping reactions, which increases the solvent exposure of H37 and D63. The proposed GTP-SAM binding mode, depicted in Fig. 5, serves as a reference point in the docking analysis of the various inhibitors in the main binding pocket.

### Mapping of resistance mutations on the nsP1 structure.

**FIG 6:**
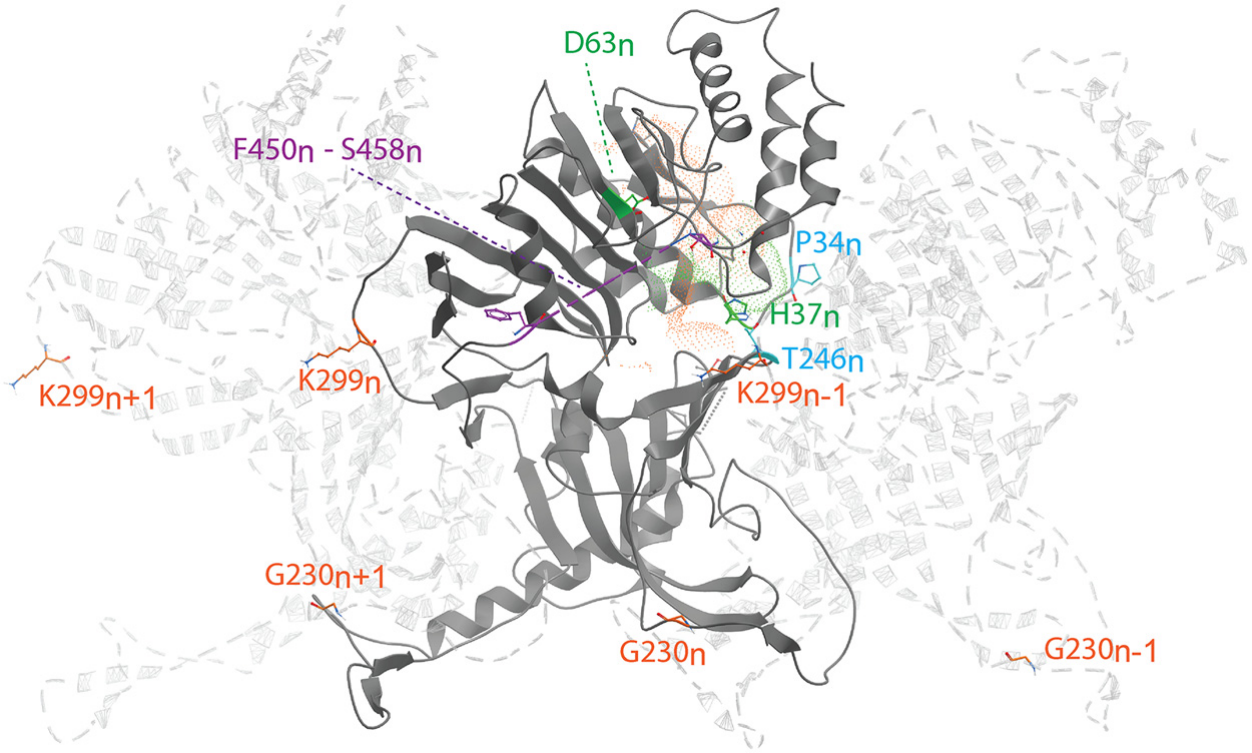
Key amino acid residues linked to resistance to CHIKV nsP1 inhibitors, mapped to the CHIKV nsP1 cryo-EM structure (PDB code 6Z0V), represented as three consecutive nsP1 protomers: n, n+1, and n–1. Residues are color-coded as follows: green, catalytic residues H37 and D63; blue, residues P34 and T246, involved in resistance to the MADTP series; orange, residues G230 and K299, conferring resistance to the FHNA series. Residues F450 to S458 (purple) flank the area where residues S454 and W456, involved in resistance to the CHVB series, would be located. Orange and green dotted volumes represent the proposed binding pockets of GTP and SAM, respectively, for reference.

Our cross-resistance studies with CHIKV nsP1 mutants revealed a pattern for resistance to various CHIKV nsP1-targeting compounds. Namely, the MADTP and CHVB series appear to have similar modes of action, while FHNA and its 3′-keto form (referred to below as the FHNA series) appear to interfere with CHIKV nsP1 function via a different mechanism. Figure 6 shows the positions of the amino acid changes responsible for resistance to these inhibitors on a graphical representation of three consecutive nsP1 protomers as part of the nsP1 dodecameric ring (28). The subscripts n+1, n, and n–1 refer to the nsP1 protomers to which the amino acid residues belong. Residue P34_n_, mutation of which causes resistance to the MADTP series, is part of the catalytic loop in the active site and delimits the GTP-binding site. Residues G230_n_ and K299_n_, which are mutated in the FHNA-resistant virus, are found in different functional regions of nsP1. Residue G230_n_ is located in membrane-binding and oligomerization (MBO) loop 1, which folds over the n–1 protomer. Residue K299_n_ lines the entrance to the SAM-binding site of the n+1 protomer. Residues G230_n_ and K299_n–1_ flank a smaller predicted binding pocket (pocket 2 in Fig. 4), which could explain the mode of action of the FHNA series and the mechanism of drug resistance. Unfortunately, the CHIKV nsP1 cryo-EM structure includes a density gap between residues 450 and 458, and therefore, the positions of residues S454 and W456, which are mutated in the CHVB-resistant virus, could not be mapped. Presumably, both S454_n_ and W456_n_ are located in the proximity of the entrance to the SAM-binding site, based on the positions of amino acids flanking this region.

### Predicted binding mode of CHIKV nsP1-targeting compounds.

**FIG 7:**
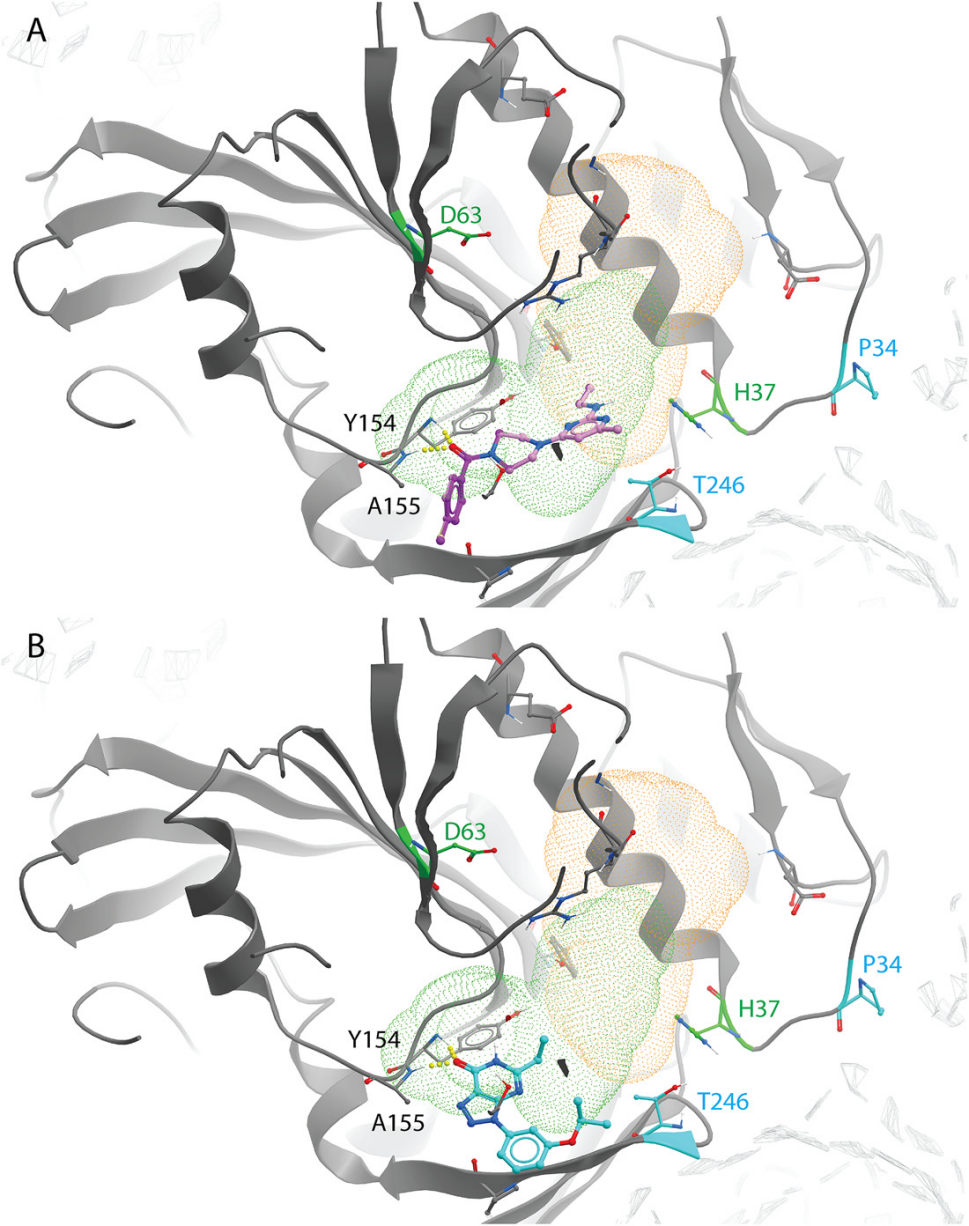
Predicted binding mode of the CHVB and MADTP series in the CHIKV nsP1 SAM-binding site of the main binding pocket (PDB code 6Z0V). (A) CHVB-066 (purple) and CHVB-032 (pink) in complex with CHIKV nsP1, occupying the SAM-binding site (green dots). Both CHVB compounds form hydrogen bonds with Y154 and A155 (in yellow) and share a binding mode. The strength of hydrogen bonds is represented by the diameter of the sphere. (B) MADTP-372 (blue) in complex with CHIKV nsP1, occupying the SAM-binding site (green dots). This compound forms hydrogen bonds with Y154 and A155 (in yellow). P34 (in blue) is the main residue responsible for MADTP compound resistance. T246 (in blue) is an additional residue that, upon mutation, causes some level of resistance to MADTP.

**FIG 8:**
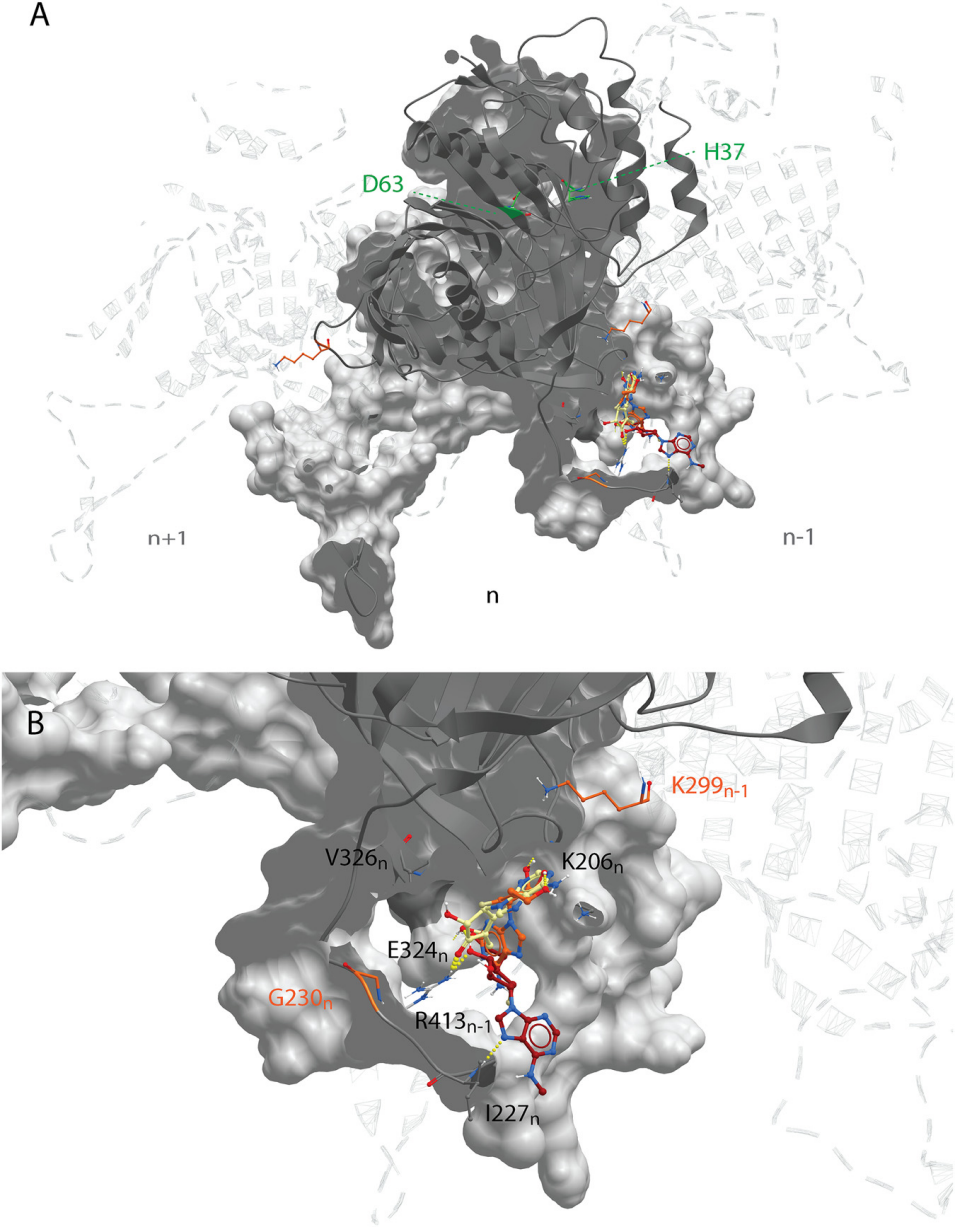
Binding mode of the FHNA series in the CHIKV nsP1 secondary binding pocket. (A) The FHNA series is suggested to bind to a secondary binding pocket (pocket 2) in the RAMBO domain of CHIKV nsP1 (PDB code 6Z0V) outside of the main binding pocket. (B) The defined binding pocket is flanked by residues G230_n_ and K299_n–1_. FHNA and FHA have similar binding modes that are not shared by the inactive analogue FMA.

Docking studies with the CHIKV nsP1 cryo-EM structure predicted that compounds of the CHVB and MADTP series bind at the SAM-binding site of the main binding pocket (Fig. 7A and B). In this model, hydrogen bonds with the backbone of Y154 and A155 seem crucial for the ligand binding of both series of compounds. Compounds of the CHVB series (CHVB-032 and CHVB-066) are predicted to bind more strongly than MADTP-372 (i.e., with lower docking scores) (Table 3). In mutagenesis experiments, it was observed that the P34S mutation and, to a lesser extent, the T246A mutation are involved in resistance to MADTP-372. Both residues form part of the catalytic site of nsP1, while only P34 is located in the catalytic loop (Fig. 6). Considering the predicted binding poses of MADTP-372 and CHVB compounds, they are unlikely to make direct interactions with P34 or T246, since the distance to these residues is too great. However, residue P34 and, to a lesser extent, T246 appear to be crucial for maintaining the conformation of the catalytic loop in the active site, and their mutations are likely to affect the activity of any small molecule binding at the main binding pocket. The S454G and W456R substitutions, which confer resistance to the compounds of the CHVB series, could not be directly mapped in the available cryo-EM structure, but they are likely positioned at the entrance of the SAM-binding site (Fig. 6). Mutations in this region of nsP1 could arise due to a compensatory effect increasing SAM binding or SAM access to the SAM-binding site. Consequently, compensatory mutations would counteract the inhibition by CHVB compounds. The SAM analogue sinefungin was also docked at the predicted main binding pocket, but its pose is not considered here, since it was expected to bind in a manner similar to that of SAM. Based on the docking results presented in Table 3, it could be that sinefungin preferentially drifts to the GTP-binding site because it is more solvent exposed than the SAM-binding site in the current CHIKV nsP1 conformation. The docking results for the FHNA series support the hypothesis that they target a different region and function of nsP1, since they preferentially bind to pocket 2 in the RAMBO domain of nsP1 (Fig. 8A). In pocket 2, the compounds of the FHNA series would form hydrogen bonds with residues close to residue G230, which is involved in resistance to this series (Fig. 8B). The inactive analogue 6′-β-fluoro-*N*^6^-methyladenosine (FMA) could also be docked in pocket 2, but it portrayed a different binding mode, providing a structural basis for the differences in antiviral activity between FHNA and the inactive analogue. In general, the CHVB and MADTP series bind with lower affinity to pocket 2 (Table 4), with the exception of CHVB-032, which seems to be stabilized both by hydrogen bonds and by hydrophobic interactions in this pocket. The difference between the docking scores of CHVB-066 and CHVB-032 could explain the higher potency of CHVB-066 in anti-CHIKV assays (Table 1), whereby CHVB-032 could be more easily sequestered in pocket 2 than its analogue CHVB-066.

**TABLE 3:** Docking results for selected poses from the ICM small-molecule docking method in the main binding pocket for the endogenous ligands GTP and SAM and for inhibitors belonging to the CHVB, MADTP, and FHNA series

Compound	Pocket^*a*^	Pose	Score^*b*^	Hbond^*c*^	Hphob^*c*^	VwInt^*c*^
GTP	GTP	1	–33.68	–19.15	–3.27	–31.4
SAM	SAM	1	–2.65	–7.75	–5.30	–21.55
Sinefungin	1 (GTP)	1	–17.35	–11.13	–4.862	–22.55
CHVB-066	1 (SAM)	1	–21.76	–5.051	–7.936	–22.71
CHVB-032	1 (SAM)	1	–21.29	–5.124	–7.414	–21.72
MADTP-372	1 (SAM)	1	–17.54	–5.711	–6.509	–22.21
FHNA	1 (GTP)	2	–14.15	–6.059	–4.099	–21.66
3′-Keto form of FHNA	1 (GTP)	1	–13.88	–7.497	–3.73	–18.34
FMA	1 (GTP)	1	–14.97	–6.949	–4.094	–21.27

a SAM and GTP in parentheses refer to the SAM- and GTP-binding sites, respectively, within the main binding pocket (pocket 1).

b Not comparable between different binding pockets.

c Hbond, Hphob, and VwInt, contributions of the hydrogen bond, hydrophobic, and van der Waals interaction networks, respectively, to the docking score.

**TABLE 4:** Docking results for selected poses from the ICM small-molecule docking method in the secondary binding pocket^*a*^ for inhibitors belonging to the CHVB, MADTP, and FHNA series

Compound	Pocket	Pose	Score^*b*^	Hbond^*c*^	Hphob^*c*^	VwInt^*c*^
Sinefungin	2	1	–8.302	–10.93	–4.45	–23.17
CHVB-066	2	1	–6.819	–0.685	–6.887	–24.27
CHVB-032	2	1	–16.51	–3.084	–5.966	–24.1
MADTP-372	2	1	–9.269	–2.374	–4.28	–22.88
FHNA	2	1	–10.26	–4.138	–3.471	–19.22
3′-Keto form of FHNA	2	2	–11.25	–8.237	–3.701	–21.76
FMA	2	1	–10.36	–5.363	–3.548	–15.02

a Pocket 2.

b Not comparable between different binding pockets.

c Hbond, Hphob, and VwInt, contributions of the hydrogen bond, hydrophobic, and van der Waals interaction networks, respectively, to the docking score.

## DISCUSSION

In this study, we have explored the cross-functional relationships of a variety of chemically distinct CHIKV nsP1 inhibitors (Fig. 1). Here, we present a model that could explain the antiviral mechanisms of the inhibitors belonging to the MADTP, CHVB, and FHNA series. Cell-based phenotypic cross-resistance assays revealed that the CHVB and MADTP series have similar modes of action that differ from that of the FHNA series. Interestingly, all of the CHIKV nsP1 mutants that were resistant to the different nsP1-targeting compounds were sensitive to sinefungin (Fig. 2). In enzymatic assays with wt SFV nsP1, CHVB-032 and CHVB-066 completely blocked the formation of the covalent [^32^P]m^7^GMP-nsP1 complex, while MADTP-372 also inhibited SFV nsP1 enzymatic activity, though less potently (Fig. 3A). Sinefungin only weakly inhibited SFV nsP1 enzymatic activity, and curiously, its addition to the assay in the absence of SAM led to the formation of a radioactive product, presumably the covalent [^32^P]m^7^GMP-nsP1 complex (Fig. 3A and B). In enzymatic assays with oligomeric wt CHIKV nsP1, the compounds CHVB-032, CHVB-066, and MADTP-372 inhibited the formation of the covalent [^32^P]m^7^GMP-nsP1 complex in a dose-dependent manner in the low micromolar range (Fig. 3C). Sinefungin was an inefficient inhibitor of CHIKV nsP1 enzymatic activity *in vitro* (Fig. 3D). FHNA was not active in this biochemical assay irrespective of the presence or absence of DTT, indicating that it does not directly inhibit the enzymatic activity of CHIKV nsP1 (Fig. 3E). Both the cell-based and enzymatic assays indicated that these CHIKV nsP1 inhibitors target different functions of the protein. To elucidate the mechanism of action of these compounds in more detail, we performed a molecular-docking study with the recently solved CHIKV nsP1 cryo-EM structure. Importantly, the molecular-docking experiments described here were performed on an enzymatically active form of CHIKV nsP1. Recently, Jones et al. reported that CHIKV nsP1 is active when assembled as oligomers into a ring-shaped membrane-associated complex (28). Using this structure, we identified two binding pockets within a single nsP1 protomer: the main binding pocket (pocket 1) in the capping domain and the secondary binding pocket (pocket 2) in the RAMBO domain (Fig. 4). The main binding pocket, which forms the catalytic site of CHIKV nsP1, is further divided into two binding sites occupied by the endogenous ligands SAM and GTP (Fig. 5). The mutations responsible for resistance to the MADTP, CHVB, and FHNA series mapped to different functional regions of CHIKV nsP1 (Fig. 6), supporting our observations from the cell-based and enzymatic assays. The molecular-docking experiments predicted that the MADTP and CHVB series bind at the SAM-binding site in the main binding pocket (Fig. 7A and B). Here, the MADTP and CHVB compounds would likely exert their inhibitory effect by competing with SAM. The differences in potency of these compounds are supported by differences in docking scores. In contrast, the compounds of the FHNA series most probably bind to a different region of nsP1, seemingly not directly disrupting the catalytic activity (Fig. 8). Instead, the FHNA series appears to interfere with the membrane binding and oligomerization of nsP1 by binding to pocket 2 in the RAMBO domain and thus might indirectly affect nsP1 function. Lastly, sinefungin does not seem to bind in a manner similar to those of the inhibitors of the MADTP, CHVB, and FHNA series. Even though sinefungin structurally resembles SAM, it preferentially docked at the GTP-binding site (Table 3). However, the docking results for sinefungin need to be interpreted with caution because the SAM’s binding pose in itself is not very reliable in the current CHIKV nsP1 conformation. As mentioned below, in Materials and Methods, SAM and sinefungin were docked differently (i.e., using different docking grids or reference residues). In addition, more experimental data would need to be obtained to explain why sinefungin stimulates GTase activity and does not serve as a competitive SAM inhibitor. In summary, we identified several classes of CHIKV nsP1 inhibitors with unique modes of action by our cell-based and biochemical assays as well as molecular-docking studies.

Furthermore, the molecular-docking experiments gave rise to interesting observations regarding the structural impact of the mutated residues in the MADTP-, CHVB- and FHNA-resistant viruses. The residues conferring resistance to the MADTP and CHVB series were found to gate the SAM-binding site in the main binding pocket (Fig. 6). Specifically, the residues that are mutated in the MADTP-resistant viruses (substitutions P34S and T246A) were located close to the catalytic residue H37. The residues that are mutated in the CHVB-resistant viruses (substitutions S454G and W456R) were predicted to be located in proximity to the SAM-binding site despite a density gap in the CHIKV nsP1 structure, which precluded precise mapping of these residues (Fig. 6). Given the predicted positions of these amino acids, both MADTP and CHVB compounds are unlikely to interact directly with these residues in the active site. The drug resistance observed could be achieved by compensatory mutations that destabilize the active site; for example, the MADTP resistance mutation P34S could affect the position of the catalytic loop in the active site, including the position of the catalytic residue H37. The ensuing conformational change of the active site could thus negatively influence the binding mode of the MADTP series. The same negative effect would be expected from mutations of other residues in the catalytic loop, for example, from alteration of the charge of residue D36. The CHVB resistance mutations also appear to be compensatory, since they are predicted to be located farther from the SAM-binding site. These mutations could facilitate SAM binding or increase SAM access to the SAM-binding site. In contrast, residues G230_n_ and K299_n–1_, which are mutated in the FHNA-resistant virus, flank pocket 2 in the RAMBO domain (Fig. 6). Residue G230 is located in MBO loop 1, involved in the formation of membrane-binding spikes, facilitating the membrane binding and assembly of oligomeric nsP1. More specifically, residue G230_n_ promotes interactions with the RAMBO MBO loop 2 of the n–1 protomer. Residue K299_n_ gates the entry to the SAM-binding site of the n+1 protomer. Interestingly, the V326M substitution, which, together with G230R, was previously implicated in resistance to difluoromethylornithine (32), also mapped to pocket 2, suggesting that compounds linked to inhibition of methionine metabolism localize to a discrete binding site within the nsP1 structure (Fig. 8). Furthermore, nsP1 oligomerization allosterically activates the enzyme; therefore, mutations that disrupt this process are expected to lead to a loss of nsP1 enzymatic activity (28). Our enzymatic assays with wt SFV nsP1 revealed that the 3′-keto form of FHNA only partially inhibited the formation of the covalent [^32^P]m^7^GMP-nsP1 intermediate (22). The rather negligible inhibitory effect of the 3′-keto form of FHNA in the SFV nsP1 covalent-complex formation assay relative to the effects of compounds belonging to the MADTP and CHVB series and the lack of inhibition by FHNA in an enzymatic assay with oligomeric wt CHIKV nsP1 could be explained by an allosteric effect on nsP1 enzymatic activity, affecting the oligomerization of nsP1. Last, but not least, FMA, which was inactive in CHIKV CPE reduction assays, showed a binding mode different from that of active FHNA, differing by the N-methylation at the N-6 position of the purine ring and the double bond on the cyclohexyl ring.

The major limitation of this study and the current model is that the available CHIKV nsP1 cryo-EM structure has no ligands in the active site. In addition, the findings by Jones et al. suggest a complex mechanism of nsP1 oligomerization and activation (28), which would be very difficult to capture with the techniques used in this study, potentially leading to discrepancies between the modeled and experimental data. Importantly, the molecular-docking experiments presented in this study were performed on the dodecameric form of CHIKV nsP1, which could lead to a potential bias when compounds are docked on monomeric nsP1. For example, MADTP-372 preferentially binds at the GTP-binding site when docked to monomeric nsP1 as opposed to oligomeric nsP1. Here, we reported on the molecular docking of CHIKV nsP1 inhibitors considering the whole CHIKV nsP1 complex, because we aimed to approximate our results to the active form of CHIKV nsP1 likely found in CHIKV-infected cells, as suggested by tomographic reconstructions of cells infected with Flock House virus (28). Furthermore, the resistance mutations described in this study were not located in the compound-docking sites in the apo nsP1 structure. Their position might further change upon conformational reorganization of nsP1, which is predicted to occur after m^7^GTP relocates in the proximity of the catalytic residue H37 in the main binding pocket to form the covalent m^7^GMP-nsP1 intermediate (28). Previous studies using recombinant SFV or VEEV nsP1 mutants expressed in E. coli defined residues important for the catalytic activity of alphavirus nsP1. For example, SFV nsP1 mutants carrying amino acid substitutions of conserved residues are either completely inactive in assays measuring both MTase and GTase activities (D64A, D90A, and C142A mutants) or had a very low level of activity (C135A and Y249A mutants) (16), suggesting an important role of these residues in RNA capping. By use of a similar assay, mutational studies with VEEV nsP1 showed that the D63A mutant was devoid of MTase activity while the H37A mutant had abrogated GTase activity (33). All of these residues are positioned at or near the active site in the current CHIKV nsP1 model. Residues H37 and Y248 line the GTP-binding site, while residue D89 is exclusively involved in SAM binding. Residues C134 and C141 are found in the Zn-binding site below the active site (28). Residue Q19 in VEEV nsP1 was identified as a key residue for MTase activity, and it was shown that a Q19K mutation modulates SAM and GTP binding (34). This residue was shown to be important for the enzymatic activity of nsP1 and would be expected to lie in the proximity of the active site. Nevertheless, the recent findings by Jones et al. describing the process of CHIKV nsP1 oligomerization into structurally novel capping rings challenge the biochemical data from these assays (28). Confirmatory experiments with oligomeric wt and mutant CHIKV nsP1 would need to be performed to validate the results obtained with other forms of alphavirus nsP1. It still remains very puzzling why compounds docking at the active site of CHIKV nsP1, including the MADTP and CHVB series and sinefungin, are not active against the related alphavirus SFV (Table 1). Previously, it was shown that FHA and FHNA, which were active against both CHIKV and SFV, were inactive against SINV (22), and the MADTP and CHVB series were also found to be inactive against other alphaviruses (21, 24). The current opinion in the field holds that the ring-shaped, membrane-associated nsP1 complex is conserved among alphaviruses. Besides its enzymatic functions, important for RNA capping, the nsP1 complex plays a key role in anchoring the replication complex (nsP1 to -4) to membranes (4). The nsP1 capping ring appears to interact with nsP4 on both the inner and outer sides of the pore (28).

Taking our findings together, this study predicts the mode of action of several CHIKV nsP1 inhibitors. We emphasize that our conclusions are based on a combined interpretation of docking poses and mutagenesis data. Nevertheless, other docking possibilities may exist, especially if different conformations of CHIKV nsP1 (i.e., other atomic structures in complex with either endogenous or targeted ligands, or monomeric nsP1) are considered. Our combined data suggest that compounds belonging to the MADTP and CHVB series likely interfere with CHIKV nsP1 functions directly via competitive or noncompetitive inhibition of SAM binding. FHNA and its 3′-keto form seem to bind outside the catalytic site occupied by SAM and GTP and likely have an indirect effect on nsP1 function, possibly by disrupting nsP1 oligomerization and membrane binding and/or through an allosteric effect on the catalytic site. Since nsP1 oligomerization stabilizes the capping domain, inhibition of this process would compromise the ability of CHIKV nsP1 to perform RNA capping. Our study demonstrates that CHIKV nsP1 is an interesting and relevant antiviral drug target that could be efficiently inhibited by compounds with different mechanisms of action. This would allow the development of combination therapy directed at this unique viral activity by combining functionally distinct nsP1 inhibitors and thus lowering the risk of emergence of antiviral drug resistance.

## MATERIALS AND METHODS

### Cells and virus strains.

Vero E6 cells were grown in Dulbecco’s modified Eagle medium (DMEM) supplemented with 8% fetal calf serum (FCS) and penicillin-streptomycin. Infection assays were performed in Eagle’s minimum essential medium (EMEM) supplemented with 2% FCS, 2 mM l-glutamine, and penicillin-streptomycin. CHIKV LS3 (GenBank accession no. KC149888) is an infectious clone-derived virus (35). CHIKV nsP1-P34S is a reverse-engineered LS3-derived mutant that is resistant to MADTP compounds (21). CHIKV nsP1-G230R+K299E is a reverse-engineered LS3-derived mutant resistant to FHA and FHNA (22). CHIKV nsP1-S454G+W456R is a reverse-engineered LS3-derived mutant resistant to CHVB-032 and CHVB-066 (24). Semliki Forest virus (SFV) strain SFV4 was used in cytopathic effect (CPE) reduction assays to assess the antiviral spectrum of nsP1 inhibitors.

### Compounds.

FHA, FHNA, and 6′-β-fluoro-*N*^6^-methyladenosine (FMA) were synthesized as described elsewhere (36). The compounds were maintained as 20 mM stock in dimethyl sulfoxide (DMSO) at 4°C and were used as described previously (22). MADTP-372 was synthesized as described elsewhere (20), and 10 mM stock solutions in DMSO were prepared and used as described previously (21). Sinefungin (SanBio) was dissolved to 50 mM in Milli-Q. Favipiravir (BOC Sciences) was dissolved to 100 mM in DMSO. CHVB-032 and CHVB-066 were synthesized as described elsewhere (23). CHVB-066 (25.4 mM) and CHVB-032 (29.1 mM) stock solutions in DMSO were prepared and used as described elsewhere (24). All compounds except FHA, FHNA, and FMA were stored at −20°C.

### CPE reduction assay.

CPE reduction assays were performed as described previously (22). Briefly, Vero E6 cells were seeded in 96-well clusters at a density of 5 × 10^3^ cells/well in DMEM supplemented with 8% FCS. The next day, the cells were incubated with serial dilutions of compounds prepared in EMEM supplemented with 2% FCS and either were infected with 50 μl/well of CHIKV (multiplicity of infection [MOI], 0.005) or SFV (MOI, 0.025) or were left uninfected. For phenotypic cross-resistance assays, a 10-times-higher MOI (0.05) was used (500 PFU/well). The SFV- and CHIKV-infected plates were incubated for 32 h and 96 h, respectively. Cell viability was measured using the 3-(4,5-dimethylthiazol-2-yl)-5-(3-carboxymethoxyphenyl)-2-(4-sulfophenyl)-2H-tetrazolium (MTS)/phenazine methosulfate (PMS) method (Promega, the Netherlands) by adding 20 μl/well of MTS reagent. The cells were incubated for 2 h, followed by fixation with 30 μl/well of 37% formaldehyde. Absorption was measured at 490 nm using an Envision plate reader (Perkin Elmer, USA). The 50% effective concentration (EC_50_), defined as the concentration of compound required to inhibit virus-induced cell death by 50%, and the 50% cytotoxic concentration (CC_50_), defined as the concentration of compound that reduced cell viability to 50% of that of untreated control cells, were determined using nonlinear regression with GraphPad Prism, v8.0.

### m^7^GMP-nsP1 covalent-complex formation assay.

The SFV nsP1 covalent m^7^GMP-nsP1 complex formation assay was performed as described previously (22). Briefly, the activity of SFV nsP1 was measured in a standard 30-μl reaction mixture containing 25 mM HEPES (pH 7.5), 5 mM DTT, 10 mM KCl, 2 mM MgCl_2_, 100 μM SAM, 0.75 mCi of [α-^32^P]GTP (3,000 Ci/mmol), and 0.5 μM wild-type (wt) SFV nsP1 or the active-site mutant SFV nsP1 D64A. The reaction mixture was incubated at 30°C for 30 min, and the reaction was stopped by adding 3 μl of 10% SDS. Assays with CHIKV nsP1 were performed in a 20-μl reaction mixture containing 25 mM HEPES (pH 7.5), 5 mM DTT, 10 mM KCl, 2 mM MgCl_2_, 0.75 mCi of GTP (3,000 Ci/mmol), and 0.5 μM wt CHIKV nsP1. Alternatively, assays were performed under nonreducing conditions by omitting DTT from the reaction mixture. All reaction mixtures containing CHIKV nsP1 were incubated at 30°C for 30 min, and reactions were stopped by adding 2 μl of 10% SDS. The reaction mixtures were mixed with 4× Laemmli sample buffer (LSB), and 10-μl samples were then separated in a 10% SDS-PAGE gel. The dried gels were placed in a cassette with a PhosphorImager screen. After overnight exposure, the ^32^P-labeled covalent m^7^GMP-nsP1 intermediate products were visualized with a Typhoon imager (Amersham).

### System preparation and CHIKV nsP1 molecular docking.

Docking was performed using ICM Pro software, v3.9-1b (Molsoft LLC, San Diego, CA) (37, 38). The apo CHIKV nsP1 cryo-EM structure representing a dodecameric ring (PDB code 6Z0V) was prepared by adding and optimizing the positions of hydrogen atoms, as well as the orientation and protonation states of histidine and cysteine residues and the orientation of glutamine and asparagine residues. “Chain A” in the cryo-EM structure was used as the main nsP1 monomeric structure (referred to as n), and chains n+1 and n–1, flanking chain n, were considered in order to acknowledge the complexed nature of active nsP1. The binding pockets of endogenous ligands GTP and SAM were defined by using their corresponding binding pocket residues as proposed by Jones et al. (28). The two ligands were docked separately, using default settings without constraints. Potential small-molecule binding pockets were identified by the ICM Pocket Finder method (30, 31), using chains n and n–1 as a starting point. Two predicted pockets, surrounded by 33 and 18 residues, respectively, were selected based on mutagenesis data. The proposed inhibitors CHVB-066, CHVB-032, MADTP-372, sinefungin, FHNA, the 3′-keto form of FHNA, and FMA were docked into the defined binding pockets with default settings and 10 poses stored for each. All ligands were routinely prepared by adding hydrogen atoms and assigning atomic charges. The docking results were analyzed in light of the available experimental data, and docking poses were selected accordingly between the top two poses based on the docking score and interaction networks.

### Statistical analysis.

Statistical analysis was performed, and figures were generated, using Graph-Pad Prism, v8.
